# Association of urinary phthalate metabolites with all-cause and cardiovascular disease mortality among adults with diabetes mellitus: National Health and Nutrition Examination Survey 2005–2014

**DOI:** 10.3389/fpubh.2023.1178057

**Published:** 2023-05-30

**Authors:** Zhihong Wang, Yao Deng, Sikang Gao, Zefang Lin, Zhixiong Zheng, Qin Fang, Meixiao Zhan, Taoping Sun, Guomin Huang, Xuyang Geng

**Affiliations:** ^1^Guangdong Provincial Key Laboratory of Tumor Interventional Diagnosis and Treatment, Zhuhai Precision Medical Center, Zhuhai People’s Hospital, Zhuhai Hospital Affiliated with Jinan University, Jinan University, Zhuhai, China; ^2^Department of Radiology, Tongji Hospital, Tongji Medical College, Huazhong University of Science and Technology, Wuhan, China; ^3^Department of Medical Affairs, Zhuhai People’s Hospital, Zhuhai Hospital Affiliated with Jinan University, Jinan University, Zhuhai, China

**Keywords:** urinary phthalate metabolites, diabetes mellitus, cardiovascular death, NHANES, mortality

## Abstract

**Background:**

The study regarding phthalate metabolites and mortality among diabetes mellitus (DM) is limited. We aimed to examine the association of urinary phthalate metabolites with all-cause and cardiovascular disease (CVD) mortality among adults with DM.

**Methods:**

This study included 8,931 adults from the National Health and Nutrition Examination Survey (NHANES) from 2005–2006 to 2013–2014. Mortality data were linked to National Death Index public access files through December 31, 2015. Cox proportional hazard models were used to estimate hazard ratios (HR) and 95% confidences (CIs) for mortality.

**Results:**

We identified 1,603 adults with DM [mean ± SE age, 47.08 ± 0.30 years; 50.5% (833) were men]. Mono-(carboxynonyl) phthalate (MCNP), mono-2-ethyl-5-carboxypentyl phthalate (MECPP), and the sum of Di (2-ethylhexyl) phthalate (DEHP) metabolites (∑DEHP) were positively associated with DM (MCNP: OR = 1.53, 95%CI = 1.16–2.01; MECPP: OR = 1.17, 95% CI = 1.03–1.32; ∑DEHP: OR = 1.14, 95% CI = 1.00–1.29). Among DM patients, mono-(3-carboxypropyl) phthalate (MCPP) was associated with a 34% (HR 1.34, 95% CI 1.12–1.61) increased risk of all-cause mortality while the HRs (95%CI) of CVD mortality were 2.02 (1.13–3.64) for MCPP, 2.17 (1.26–3.75) for mono-(2-ethyl-5-hydroxyhexyl) phthalate (MEHHP), 2.47 (1.43–4.28) for mono-(2-ethyl-5-oxohexyl) phthalate (MEOHP), 2.65 (1.51–4.63) for MECPP, and 2.56 (1.46–4.46) for ∑DEHP, respectively.

**Conclusion:**

This study is an academic exploration of the association between urinary phthalate metabolites and mortality among adults with DM, suggesting that exposure to phthalates might be associated with an increased risk of all-cause and CVD mortality in DM. These findings suggest that patients with DM should carefully use plastics products.

## 1. Introduction

Type 2 diabetes is one of the most common chronic diseases. The International Diabetes Federation (IDF) estimated that there were almost 537 million people with diabetes worldwide in 2021, and it was predicted to increase to 783 million by 2045 (1), which leads to a wide spectrum of healthcare expenditures and disease burdens. Simultaneously, multiple studies indicated diabetes mellitus (DM) was significantly associated with increased mortality (2, 3). The reason for excess mortality of DM could be caused by an increased risk of cardiovascular disease (CVD) (4, 5), and cardiovascular death is an important component of the excess mortality of DM patients. Pathological studies show that hyperglycemia and insulin resistance lead to vascular inflammation, vasoconstriction, thrombosis, and further atherogenesis by increasing oxidative stress, disrupting protein kinase C signaling, and other pathways (6). The prevention of cardiovascular death becomes especially vital for DM patients. However, except for the reason cardiovascular death is caused by basic pathological changes including hyperglycemia and insulin resistance in DM patients, other environmental factors also contribute to the cardiovascular death of DM patients.

Phthalates (also named phthalate acid esters) are a class of chemicals, as plasticizers or solvents widely, used in various consumer products such as food packaging, medical devices, toiletries, cosmetics, nail polish, and flooring (7, 8). With the increasing use of plastic, human is widely exposed to environmental phthalates through food, water, air and everywhere (9, 10). Phthalates play adverse roles in the human body through different mechanisms including nuclear hormone receptors (estrogen receptors, androgen receptors), membrane and nonsteroid receptors (11). The major studies showed phthalates as Endocrine Disrupting Chemicals (EDCs) significantly increased the risk of type 2 diabetes and cardiovascular disease (9, 10, 12). Sturgeon et al. indicated that no association between cardiovascular disease mortality and individual urinary phthalate metabolites was observed in the general population. However, whether phthalates would increase the risk for all-cause and cardiovascular mortality in DM patients via modification of potential shared biological pathways remains uncertain.

Evidence for the potential effects of phthalates in DM patients is still limited. It is worth noting that phthalates are primarily excreted in the urine and blood-based laboratory assays exist the potential for contamination (13), therefore the urinary concentrations of phthalate metabolites provide an excellent biomarker of exposure (9). Updated data were used from the US National Health and Nutrition Examination Survey (NHANES) 2005–2014, we explored the relationships between all-cause and cardiovascular mortality with phthalates in DM patients by assessing the differences in the urinary concentrations of phthalate metabolites.

## 2. Materials and methods

### 2.1. Study population and design

This study was performed by using NHANES datasets, which were designed to assess the health and nutritional status of adults and children in the United States with a multistage, stratified, probability sampling method (14). From 1999, NHANES became a continuous program with 2 years per cycle including interviewers, physical examinations and laboratory detecting, and almost 10 thousand representative persons participated in every cycle. Urinary phthalates metabolites were measured in a one-third subsample of individuals 6 years and older. We used five of the cycles from 2005–2006 to 2013–2014 and 16,514 participants underwent urinary phthalates testing in the five NHANES cycles. We excluded participants who were missing 12 interested phthalates metabolites laboratory data (*n* = 3,192) and were younger than 20 years old (*n* = 4,391). Finally, a total of 8,931 subjects were included in our cohort study. NHANES was conducted under the approval of the National Center for Health Statistics (NCHS) Research Ethics Review Board (ERB) and at the same time, the participants provided written informed consent to participate in the survey (15).

### 2.2. Measurement of urinary phthalate metabolites

Phthalate metabolites were measured in spot urine samples were collected during participants’ examination at the Mobile Examination Center (MEC) and stored at −20°C. Then the samples were shipped to National Center for Environmental Health, Centers for Disease Control and Prevention (CDC) for analysis. Urine samples were processed using glucuronide enzymatic digestion and the phthalate monoesters were coupled by solid phase extraction (SPE). Finally, quantitative detection of urine phthalate metabolites was conducted using high-performance liquid chromatography-electrospray ionization-tandem mass spectrometry (HPLC-ESI-MS/MS) (16). Urinary creatinine was also measured to adjust urinary dilution for each urine sample.

We focused on 12 phthalate metabolites from eight parent compounds in five cycles (from 2005–2006 to 2013–2014). As shown in Table 1, except mono(2-ethylhexyl) phthalate (MEHP) and mono-isononyl phthalate (MiNP), the other metabolites, including mono-(carboxynonyl) phthalate (MCNP), mono-(carboxyoctyl) phthalate (MCOP), mono-n-butyl phthalate (MnBP), mono-ethyl phthalate (MEP), mono-benzyl phthalate (MBzP), mono-(3-carboxypropyl) phthalate (MCPP), mono-(2-ethyl-5-hydroxyhexyl) phthalate (MEHHP), mono-(2-ethyl-5-oxohexyl) phthalate (MEOHP), mono-isobutyl phthalate (MiBP) and mono-2-ethyl-5-carboxypentyl phthalate (MECPP), were detectable in more than 95% of all participants in the cohort. Furthermore, we calculated the molar sum of Di (2-ethylhexyl) phthalate (DEHP) metabolites (∑DEHP) to estimate the total exposure of the parent phthalate DEHP. The formula is ∑DEHP (μmol/L) = (MEHP/278.34) + (MEHHP/294.34) + (MEOHP/292.33) + (MECPP/308.33) (17, 18). The concentrations of phthalates were adjusted by the urinary creatinine concentration to correct for urinary dilution, creatinine adjustment was conducted by dividing the urinary chemical concentration with the urinary creatinine concentration (Ucr) of each spot urine sample. The levels < limit of detection (LOD) was replaced by LOD/Sqrt (2).

**Table 1:** Sample-weighted, creatinine-standardized urinary phthalate metabolite concentrations in the U.S. DM and non-DM population, NHANES 2005–2014 (μg/g creatinine).

Metabolite	Parent compound	≥LOD (%)	Geometric mean		*p-*value
			DM	non-DM	
Mono-(carboxynonyl) Phthalate (MCNP)	DiDP (Di-isodecyl phthalate)	95.1	2.76	2.65	0.046
Mono-(carboxyoctyl) Phthalate (MCOP)	DiNP (Di-isononyl phthalate)	98.0	12.16	11.27	0.017
Mono-n-butyl Phthalate (MnBP)	DBP (Di-butyl phthalate)	97.6	12.94	12.31	0.291
Mono-ethyl Phthalate (MEP)	DEP (Diethyl phthalate)	99.9	73.54	65.95	0.090
Mono(2-ethylhexyl) Phthalate (MEHP)	DEHP (Di(2-ethylhexyl) phthalate)	67.3	1.56	1.93	﹤0.001
Mono-isononyl Phthalate (MiNP)	DiNP (Di-isononyl phthalate)	31.8	1.04	1.15	0.029
Mono-benzyl Phthalate (MBzP)	BzBP (Benzylbutyl phthalate)	97.8	5.15	5.41	0.153
Mono-(3-carboxypropyl) Phthalate (MCPP)	DOP (Di-n-octyl phthalate)	95.2	2.57	2.43	0.105
Mono-(2-ethyl-5-hydroxyhexyl) Phthalate (MEHHP)	DEHP (Di(2-ethylhexyl) phthalate)	99.6	12.82	12.54	0.065
Mono-(2-ethyl-5-oxohexyl) Phthalate (MEOHP)	DEHP (Di(2-ethylhexyl) phthalate)	99.0	7.98	7.68	0.016
Mono-isobutyl Phthalate (MiBP)	DiBP (Di-butyl phthalate)	97.8	6.45	6.25	0.335
Mono-2-ethyl-5-carboxypentyl Phthalate (MECPP)	DEHP (Di(2-ethylhexyl) phthalate)	99.8	20.47	19.39	0.011
DEHP (Di(2-ethylhexyl) phthalate), μmol/g		100	0.15	0.14	0.027

DM, diabetes mellitus; NHANES, National Health and Nutrition Examination Survey; LOD, limit of detection.

### 2.3. Definition of diabetes and covariates

Diabetes was defined by self-reported diagnosis by a doctor, or self-reported taking insulin/hypoglycemic medications to lower glucose, or level of hemoglobin A1c of 6.5% or greater, or level of fasting glucose of 126 mg/dL or greater, or level of two-hour glucose of 200 mg/dL or greater (19). The participants’ demographic details included age, sex (male, or female), race/ethnicity (non-Hispanic white, non-Hispanic black, Mexican American, or other), educational level (0–11, 12, or > 12 years of education), income level (poverty income, middle income, or high income) based on Poverty Impact Ratio (RIP) were self-reported. Smoking status was separated into 3 groups including never smokers (defined as people who reported that they had not smoked as many as 100 cigarettes in their lifetime), former smokers (defined as people who had smoked 100 cigarettes or more lifetime but did not smoke cigarettes currently), and current smokers (defined as people who reported that they currently smoked cigarettes every day or some days); alcohol consumption status was categorized as never drinkers and current drinkers based on whether people who drank alcohol at least 12 times in the previous year; physical activity status was defined by the times of physical activity per week (≥1 time/week as Yes, <1 time/week as No); body mass index (BMI) was calculated as weight in kilograms divided by height in meters squared (kg/m^2^) and participants were separated into underweight or normal weight (<25 kg/m^2^), overweight (25–30 kg/m^2^), and obese (≥30 kg/m^2^); hypertension status was defined by self-reported diagnosis, or use of antihypertensive drugs, or systolic blood pressure >140 mmHg, or diastolic blood pressure >90 mmHg. Total serum cholesterol level as a continuous covariate was routinely measured in the laboratory.

### 2.4. Mortality

Mortality data were linked to National Death Index public access files to determine mortality status and cause of death in a mortality follow-up through December 31, 2015. International Classification of Diseases-10th revision (ICD-10) was used to code the cause of death. The primary outcomes were all-cause and cardiovascular mortality in our study. Cardiovascular deaths were defined as ICD-10 codes I00 to I09, I11, I13, and I20 to I51.

### 2.5. Statistical analysis

The complex sampling design and weights was adapted to our statistical analyses, which was recommended by NHANES, we calculated the weighted means ± standard error (SE) for continuous variables and frequencies (weighted percentages) for categorical variables to describe the distributions of demographic characteristics and partly laboratory indicators by the DM status. In this study, phthalate metabolite levels were corrected by urinary creatinine concentrations to adjust urinary dilution. Weighted geometric means (GMs) was used to describe the distributions of phthalate metabolites corrected by urinary creatinine. We categorized all participants into low-level, middle-level and high-level based on the tertile of each urinary metabolite.

Multivariable logistic regression models were used to calculate odds ratios (ORs) and 95% confidence intervals (CIs) to assess the prevalence rates of DM associated with urinary phthalate metabolites, with models adjusted for sex, age, ethnicity group, education, family income, the survey cycle, smoking status, drinking status, physical activity, BMI, hypertension, total cholesterol, and family history of cardiovascular diseases. Cox proportional hazards regressions were used to estimate the hazard ratios (HRs) and 95% CI for cardiovascular mortality by adjusting for covariates (including age, sex, total cholesterol, BMI, race/ethnicity, smoking status, drinking alcohol status, physical activity, education level, income level, and hypertension status) in participants with DM and participants with non-DM, respectively. We estimated the adjusted HRs of ln-transformed values of each urinary phthalate metabolite concentration for CVD mortality in three models. For model 1, we performed the Cox regression adjusting for sex, age, and ethnicity group; for model 2, we performed the Cox regression adjusting for education, family income, the survey cycle, smoking status, drinking status, physical activity, and covariates in model 1; for model 3, we performed the Cox regression adjusting for BMI, hypertension, total cholesterol, family history of CVD, and covariates in model 2.

All statistical analyses were carried out by the “survey” R package in R 4.0.3 software, and with the two-sided significant level at 0.05.

## 3. Results

### 3.1. Characteristics of the study participants

The general characteristics of participants are summarized in Table 2. In this study, a total of 8,931 participants were included, of whom 1,603 were DM and 7,328 were non-DM. The mean (SE) age was 47.08 (0.30) years [59.78 (0.44) for DM and 45.08 (0.32) years for non-DM]. Among all participants, the proportion of males was 48.1% (50.5% for DM and 47.7% for non-DM). The obesity rate for all participants was 35.3, 61.3% for people with DM, and 31.3% for people with non-DM. The proportion of people who exercise regularly was 54.3% for all participants, 39.8% for people with DM, and 56.6% for people with non-DM. More than half of participants with diabetes had hypertension. In summary, compared to participants with non-DM, individuals with DM were older and more likely to be obese, had less physical activity and more people had hypertension.

**Table 2:** Demographics and clinical characteristics of participants based on diabetes status.

	All participants	Diabetes mellitus	Non-diabetes mellitus
Total number	8,931	1,603	7,328
Age, years, mean ± SE	47.08 ± 0.30	59.78 ± 0.44	45.08 ± 0.32
Sex, male, *n* (%)	4,354 (48.1)	833 (50.5)	3,521 (47.7)
Total serum cholesterol, mg/dL, mean ± SE	194.50 ± 0.57	187.53 ± 1.58	195.61 ± 0.61
**Body mass index, *n* (%)**			
Normal weight and underweight (<25 kg/m^2^)	2,638 (31.5)	230 (13.1)	2,408 (34.3)
Overweight (25–30 kg/m^2^)	2,949 (33.2)	451 (25.6)	2,498 (34.4)
Obesity (≥30 kg/m^2^)	3,219 (35.3)	877 (61.3)	2,342 (31.3)
**Race/ethnicity, *n* (%)**			
Non-Hispanic white	4,010 (68.5)	620 (65.4)	3,390 (68.9)
Non-Hispanic black	1937 (11.4)	434 (15.4)	1,503 (10.8)
Mexican-American	1,371 (8.2)	278 (8.4)	1,093 (8.2)
Others	1,613 (11.9)	271 (10.8)	1,342 (12.1)
**Smoking status, *n* (%)**			
Current	1913 (21.8)	282 (17.6)	1,631 (22.2)
Former	2,150 (24.1)	551 (34.3)	1,599 (22.5)
Never	4,861 (54.3)	769 (48.2)	4,092 (55.3)
**Drinking status, *n* (%)**			
Current	3,854 (45.3)	467 (62.4)	3,387 (42.5)
Never	4,208 (54.7)	1,003 (37.6)	3,205 (57.5)
**Physical activity, *n* (%)**			
Yes	4,243 (54.3)	556 (39.8)	3,687 (56.6)
No	4,688 (45.7)	1,047 (60.2)	3,641 (43.4)
**Educational attainment, years, *n* (%)**			
0–11	2,373 (18.1)	600 (26.5)	1,773 (16.8)
12	2,090 (23.3)	375 (25.9)	1,715 (22.9)
≥12	4,457 (58.6)	625 (47.6)	3,832 (60.3)
**Income strata size, *n* (%)**			
Poverty income (PIR ≤ 1)	1,801 (14.9)	340 (16.0)	1,461 (14.8)
Middle income (PIR > 1 & < 4)	4,232 (48.8)	807 (53.3)	3,425 (48.1)
High income (PIR ≥ 4)	2,129 (36.3)	300 (30.6)	1,829 (37.1)
**Hypertension, *n* (%)**			
Yes	3,296 (34.6)	1,056 (66.9)	2,240 (29.5)
No	5,318 (65.4)	498 (33.1)	4,820 (70.5)

PIR, Price-to-Income Ratio; Data are presented as *n* (%) for categorical data, mean (SE) for parametrically distributed data.

### 3.2. Level of sample-weighted, creatinine-standardized urinary phthalate metabolite

The detectable percentage of sample-weighted, creatinine-standardized urinary phthalate metabolites ≥LOD, GMs of 12 urinary phthalate metabolites are presented in Table 1. Except for MEHP and MiNP, the weighted proportion of levels above LOD accounted for more than 95% for each analyte, suggesting that it was detected in most participants. Next, we focused on the 10 phthalate metabolites and DEHP. We observed that participants with diabetes had higher concentrations of urinary phthalate metabolites (excluding MBzP) compared with participants without diabetes.

### 3.3. Associations of urinary phthalates with diabetes mellitus

The associations of ten urinary phthalate metabolites and DEHP with DM are given in Table 3. The results showed that MECPP and DEHP were significantly positively associated with DM. When the concentrations of MECPP and DEHP were used as continuous variables, with the increase per unit of ln-transformed urinary MECPP, the risk of DM increased by 17% (OR = 1.17, 95% CI = 1.03–1.32, *p* = 0.014) after adjusting for the covariates. Similarly, with the increase per unit of ln-transformed urinary DEHP, the risk of DM increased by 14% (OR = 1.14, 95% CI = 1.00–1.29, *p* = 0.043) after adjusting for the covariates. Compared with participants in the first tertile of MCNP, we observed participants in the third tertile of MCNP had a higher risk of DM (OR = 1.53, 95%CI = 1.16–2.01, *p* = 0.003).

**Table 3:** Associations of phthalates concentrations with DM in U.S. participants.

	DM *N* (%)	non-DM *N* (%)	OR (95% CI)	*p*-value
MCNP^*^, μg/g			1.10 (0.98–1.24)	0.105
T1 (<1.73)	572 (12.2)	2,792 (87.2)	1.00 (ref.)	
T2 (1.73–3.51)	512 (13.1)	2,360 (86.9)	1.32 (0.95–1.84)	0.092
T3 (≥3.51)	456 (15.0)	2,043 (85.0)	1.53 (1.16–2.01)	0.003
MCOP^*^, μg/g			1.06 (0.96–1.17)	0.232
T1 (<5.55)	528 (12.0)	2,778 (88.0)	1.00 (ref.)	
T2 (5.55–18.27)	536 (13.8)	2,335 (86.2)	1.09 (0.77–1.52)	0.628
T3 (≥18.27)	476 (14.5)	2,082 (85.5.9)	1.19 (0.86–1.65)	0.289
MnBP^*^, μg/g			0.98 (0.83–1.15)	0.813
T1 (<8.93)	485 (13.1)	2,396 (86.9)	1.00 (ref.)	
T2 (8.93–17.40)	537 (13.0)	2,454 (87.0)	0.93 (0.68–1.28)	0.658
T3 (≥17.40)	518 (14.2)	2,345 (85.8)	0.97 (0.67–1.40)	0.875
MEP^*^, μg/g			1.05 (0.97–1.14)	0.243
T1 (<31.82)	412 (12.6)	2,090 (87.4)	1.00 (ref.)	
T2 (31.82–108.75)	477 (13.3)	2,310 (86.7)	1.34 (0.89–2.00)	0.156
T3 (≥108.75)	651 (14.2)	2,795 (85.8)	1.28 (0.88–1.85)	0.194
MBzP^*^, μg/g			0.94 (0.84–1.07)	0.358
T1 (<3.57)	599 (13.9)	2,682 (86.1)	1.00 (ref.)	
T2 (3.57–7.98)	555 (13.4)	2,435 (86.3)	0.86 (0.63–1.19)	0.363
T3 (≥7.98)	386 (12.3)	2,078 (87.7)	0.88 (0.64–1.22)	0.447
MCPP^*^, μg/g			1.05 (0.94–1.17)	0.385
T1 (<1.49)	552 (12.7)	2,745 (87.3)	1.00 (ref.)	
T2 (1.49–3.33)	560 (13.3)	2,478 (86.7)	1.17 (0.84–1.62)	0.350
T3 (≥3.33)	428 (14.3)	1,972 (85.7)	1.15 (0.83–1.58)	0.391
MEHHP^*^, μg/g			1.11 (0.99–1.25)	0.084
T1 (<7.50)	476 (11.7)	2,579 (88.3)	1.00 (ref.)	
T2 (7.50–16.80)	562 (15.0)	2,438 (85.0)	1.26 (0.84–1.89)	0.259
T3 (≥16.80)	502 (13.5)	2,178 (86.5)	1.34 (0.91–1.99)	0.140
MEOHP^*^, μg/g			1.12 (1.00–1.27)	0.056
T1 (<4.63)	464 (12.0)	2,627 (88.0)	1.00 (ref.)	
T2 (4.63–10.00)	560 (13.8)	2,440 (86.2)	1.05 (0.75–1.47)	0.783
T3 (≥10.00)	516 (14.5)	2,128 (85.5)	1.24 (0.87–1.76)	0.236
MiBP^*^, μg/g			1.07 (0.93–1.23)	0.345
T1 (<4.58)	470 (13.0)	2,389 (87.0)	1.00 (ref.)	
T2 (4.58–8.70)	506 (12.7)	2,435 (87.3)	0.93 (0.67–1.28)	0.649
T3 (≥8.70)	564 (14.6)	2,371 (85.4)	1.21 (0.91–1.61)	0.175
MECPP^*^, μg/g			1.17 (1.03–1.32)	0.014
T1 (<11.97)	479 (12.0)	2,637 (88.0)	1.00 (ref.)	
T2 (11.97–25.68)	554 (14.3)	2,449 (85.7)	1.21 (0.83–1.75)	0.313
T3 (≥25.68)	507 (14.1)	2,109 (85.9)	1.39 (0.99–1.97)	0.057
∑DEHP^*^, μmol/g			1.14 (1.00–1.29)	0.043
T1 (<0.09)	462 (12.0)	2,541 (88.0)	1.00 (ref.)	
T2 (0.09–0.19)	574 (14.1)	2,531 (85.9)	1.10 (0.74–1.63)	0.646
T3 (≥0.19)	504 (14.2)	2,123 (85.8)	1.38 (0.94–2.02)	0.095

DM, diabetes mellitus; OR, odds ratio; CI, confidence interval.

Adjusted for sex, age, ethnicity group, education, family income, the survey cycle, smoking status, drinking status, physical activity, BMI, hypertension, total cholesterol, and family history of cardiovascular diseases.

### 3.4. Associations of phthalates with all-cause and CVD mortality in participants with DM and non-DM

Next, for exploring the harm of phthalates, we performed Cox proportional hazards regressions to access whether urinary phthalate metabolites were related to all-cause and cardiovascular mortality in participants with DM. Table 4 presents hazard rate estimates of all-cause and cardiovascular mortality for 10 urinary phthalate metabolites and ∑DEHP in participants with DM. Based on the model 1 after adjustment for sex, age and ethnicity group, MEOHP was statistically significantly increased cardiovascular mortality (HR = 1.45, 95%CI = 1.02–2.06, *p* = 0.037). After additional adjustment including education, family income, the survey cycle, smoking status, drinking status and physical activity (model 2), MCPP and MECCP significantly increased the risk of cardiovascular mortality by 66 and 60%, respectively (HR = 1.66, 95%CI = 1.08–2.56, *p* = 0.020 and HR = 1.60, 95%CI = 1.01–2.55, *p* = 0.047). Based on the most fully adjusted model 3, urinary of MCPP was also observed statistically significantly increased cardiovascular mortality (HR = 2.02, 95%CI = 1.13–3.64, *p* = 0.018), and the similar associations were also observed in MEHHP (HR = 2.17, 95%CI = 1.26–3.75, *p* = 0.006), MEOHP (HR = 2.47, 95%CI = 1.43–4.28, *p* = 0.001), MECPP (HR = 2.65, 95%CI = 1.51–4.63, *p* = 0.001). In addition, MCPP also increased the risk of all-cause mortality by 34% (HR = 1.34, 95%CI = 1.12–1.61, *p* = 0.001). ∑DEHP is the molar sum of four metabolites (MEHP, MEHHP, MEOHP and MECCP). After adjusting for the covariates, ∑DEHP was significantly positively associated with the hazard of cardiovascular mortality in participants with DM (HR = 2.56, 95%CI = 1.46–4.46, *p* = 0.001). In addition, we did not find evidence for associations between others urinary phthalate metabolites and mortality in participants with DM. We also analyzed the relationship phthalate metabolites with all-cause and CVD mortality in participants with non-DM based on the most fully adjusted model 3, no significant associations were detected (Supplementary Table S1).

**Table 4:** Associations of phthalate concentrations with all-cause and cardiovascular disease mortality among participants with DM.

	All-cause mortality of HR (95% CI) among DM	*p*-value	Cardiovascular disease mortality of HR (95% CI) among DM	*p*-value
**MCNP** ^*^ **, μg/g**				
Model 1	1.08 (0.90–1.30)	0.421	1.28 (0.76–2.15)	0.356
Model 2	1.11 (0.87–1.42)	0.393	1.69 (0.84–3.40)	0.144
Model 3	1.12 (0.84–1.48)	0.444	1.37 (0.49–3.83)	0.552
MCOP^*^, μg/g				
Model 1	0.93 (0.82–1.05)	0.230	0.81 (0.57–1.13)	0.209
Model 2	1.07 (0.89–1.230)	0.464	1.04 (0.57–1.89)	0.897
Model 3	1.22 (0.97–1.54)	0.083	0.98 (0.47–2.06)	0.958
MnBP^*^, μg/g				
Model 1	1.16 (0.96–1.40)	0.120	1.35 (0.86–2.12)	0.190
Model 2	1.11 (0.91–1.36)	0.315	1.37 (0.82–2.26)	0.219
Model 3	1.22 (0.97–1.54)	0.095	1.58 (0.51–4.92)	0.431
MEP^*^, μg/g				
Model 1	1.02 (0.93–1.12)	0.725	0.94 (0.69–1.28)	0.712
Model 2	0.99 (0.88–1.12)	0.853	0.87 (0.66–1.14)	0.298
Model 3	1.01 (0.86–1.18)	0.918	1.24 (0.84–1.83)	0.290
MBzP^*^, μg/g				
Model 1	1.17 (0.95–1.44)	0.131	1.21 (0.80–1.84)	0.367
Model 2	1.18 (0.97–1.43)	0.100	1.26 (0.88–1.81)	0.213
Model 3	1.28 (0.95–1.73)	0.110	1.25 (0.53–2.97)	0.606
MCPP^*^, μg/g				
Model 1	0.98 (0.84–1.16)	0.843	1.16 (0.80–1.68)	0.436
Model 2	1.15 (0.95–1.38)	0.147	1.66 (1.08–2.56)	0.020
Model 3	1.34 (1.12–1.61)	0.001	2.02 (1.13–3.64)	0.018
MEHHP^*^, μg/g				
Model 1	1.16 (0.99–1.34)	0.067	1.40 (0.96–2.04)	0.077
Model 2	1.16 (0.96–1.41)	0.119	1.43 (0.87–2.36)	0.159
Model 3	1.21 (0.97–1.52)	0.086	2.17 (1.26–3.75)	0.006
MEOHP^*^, μg/g				
Model 1	1.13 (0.94–1.35)	0.180	1.45 (1.02–2.06)	0.037
Model 2	1.16 (0.94–1.44)	0.163	1.55 (0.94–2.54)	0.083
Model 3	1.23 (0.98–1.56)	0.079	2.47 (1.43–4.28)	0.001
MiBP^*^, μg/g				
Model 1	1.00 (0.83–1.21)	0.998	0.88 (0.64–1.21)	0.419
Model 2	0.98 (0.78–1.22)	0.832	0.87 (0.61–1.26)	0.465
Model 3	1.00 (0.74–1.35)	0.986	0.84 (0.36–1.94)	0.678
MECPP^*^, μg/g				
Model 1	1.16 (0.98–1.37)	0.086	1.46 (0.99–2.16)	0.059
Model 2	1.22 (1.00–1.49)	0.053	1.60 (1.01–2.55)	0.047
Model 3	1.20 (0.94–1.53)	0.143	2.65 (1.51–4.63)	0.001
∑DEHP^*^, μmol/g				
Model 1	1.16 (0.98–1.38)	0.077	1.46 (0.99–2.15)	0.054
Model 2	1.20 (0.98–1.46)	0.084	1.56 (0.96–2.54)	0.075
Model 3	1.22 (0.96–1.54)	0.110	2.56 (1.46–4.46)	0.001

DM, diabetes mellitus; HR, hazard ratio; CI, confidence interval.

Model 1, adjusted for sex, age, and ethnicity group.

Model 2, adjusted for education, family income, the survey cycle, smoking status, drinking status, physical activity, and covariates in model 1.

Model 3, adjusted for BMI, hypertension, total cholesterol, and family history of cardiovascular diseases, and covariates in model 2.

## 4. Discussion

We found that phthalate metabolites were associated with high prevalent DM in the cross-sectional study among U.S. adults. Notably, in the prospective cohort analysis of U.S. adults, we also found that higher concentrations of urinary levels of phthalate metabolites, especially MCPP, MEHHP, MEOHP, MECPP and ∑DEHP, were significantly associated with an increased risk of CVD mortality in participants with diabetes. Moreover, MCPP also was associated with an increased risk of all-cause mortality. The association was independent of traditional risk factors, including BMI, hypertension, total cholesterol and family history of cardiovascular disease. The ∑DEHP and three of four DEHP oxidative metabolites (MEHHP, MEOHP, MECPP) were consistent with an increased risk of CVD mortality in our results. This major finding indicated that the oxidative phthalates metabolites of DEHP might serve as better predictors for cardiovascular mortality in participants with diabetes.

Phthalate metabolites can affect the homeostasis of lipids and glucose, leading to insulin resistance, and thereby increasing the risk of diabetes and CVD (20). This is consistent with our findings that phthalate metabolites are associated with high prevalence of diabetes. In addition, it has been found that exposure to phthalates may reduce insulin levels in fetal rats (21), which may lead to insulin resistance in adulthood (22). In addition to insulin resistance, the studies reported phthalate metabolites are associated with risk factors for cardiovascular mortality, including weight change (23), blood glucose (24), and metabolic syndrome (25). Thus, diabetes patients might be more susceptible to an increase in risk of cardiovascular mortality due to phthalate metabolites.

Previous epidemiological studies and experimental animal research have shown that urinary phthalate metabolites are associated with an increased risk of CVD events and mortality (10, 26, 27). A cross-sectional study based on two cycles of NHANES survey data (2001–2004) reported that MCPP and MnBP metabolites were associated with a higher risk of stroke after being fully adjusted from the general population (28). However, in another prospective cohort analysis including 5,080 participants, Sturgeon et al. (13) found that urinary levels of phthalate metabolites were not associated with increased cardiovascular mortality by comparing hazard ratios in the highest and lowest quartiles based on the NHANES 1999–2008. The association of phthalate metabolites and cardiovascular mortality is still controversial, which could be because observational studies are susceptible to uncontrolled confounding. In addition, current studies were conducted among general populations and did not perform analysis in participants with DM. Among patients with diabetes, evidence is limited regarding the potential health damage of phthalate metabolites, particularly about mortality. For example, among 675 Chinese adults with diabetes, Zhang et al. (29) observed that MEP and MiBP were positively associated with CVD. Moreover, the study did pre-specified CVD as the primary endpoint and was only tested in the Chinese population. In our cohort study using a nationally representative sample of U.S. adults with diabetes, we found urinary levels of phthalate metabolites are associated with all-cause and CVD mortality after multivariate adjustment, even though the association was independent of traditional risk factors. Moreover, the oxidative metabolites MEHHP, MEOHP and MECPP are the main metabolites and suitable biomarkers for exposure to this compound (30), so our results are more detailed and convincing. More large prospective and experimental studies are needed to confirm these findings.

Although underlying mechanism of the observed association between phthalates and cardiovascular mortality in individuals with DM remains to be elucidated. Phthalate metabolites exposure may alter the signaling pathways of cells responsible for lipid metabolism and balance which can result in lipid accumulation and possibly be susceptibility to CVD (10, 31). Previous studies had suggested that DEHP harms the function of chicken (32) and rat (33) embryonic cardiomyocytes. Oxidative stress also has been considered as a possible mechanism for phthalates to cause cardiometabolic risk. Studies have reported the association between urinary phthalate metabolites and oxidative stress (16, 34). In another study among 329 China adults with diabetes, they found phthalates exposure was associated with oxidative stress in participants with diabetes, but there was not comparable with the general population, and further toxicology studies are needed (35). Among 300 participants with diabetes over the age of 50 in Shanghai, Dong et al. (36) found that the phthalate exposure was a positive association with γ-glutamiltransferase and oxidative stress biomarkers (8-hydroxy-2′-deoxyguanosine and malondialdehyde) and could induce cardiometabolic risk in serum and the risk of insulin resistance by measured 10 phthalate metabolites in urine and biomarkers of oxidative stress. Nevertheless, more mechanistic studies are needed to further elucidate the underlying mechanisms through which urinary phthalate metabolites as predictors of CVD mortality among participants with diabetes.

Given the pervasiveness of phthalates in our environment, it is important to fully understand their potential impact on health. This study has several strengths, the study includes the prospective cohort study design, and our findings from the nationally representative dataset that urinary phthalate metabolites might be related to cardiovascular mortality in participants with DM, which facilitates recapitulation of our findings and control for potential confounders. In addition, the use of NHANES data and appropriate weighting procedures can allow research results to be generalized to the Hispanic, white and black non-institutional populations in the U.S. adults. Furthermore, the variability of phthalate exposure may change with changes in personal care products, daily activities, or diets (37), we study used urinary measurements, which generally have higher concentrations of phthalate metabolites compared with serum (38) and have been shown to be a favorable biomarker of long-term exposure to phthalates (39), so more phthalate metabolites could be accurately and precisely quantified above the lower limit of detection.

Although have demonstrated a positive association between phthalate metabolites and cardiovascular mortality in the nationally representative sample of U.S. adults with DM. There are also several limitations of our study. First, urinary phthalate metabolites were measured in a single-point sample, the level of phthalate metabolites can change in a relatively short period, which may not accurately represent an individual’s typical exposure. Secondly, the confounding effects caused by psychosocial factors, genetic susceptibility, unknown confounding or accidental in the current study could not be excluded. Finally, further experimental studies are needed to clarify the mechanism by which phthalate metabolites may predict cardiovascular mortality in participants with diabetes.

## 5. Conclusion

In summary, our study is the first prospective study to explore the association between phthalate metabolites and cardiovascular mortality in U.S. adults with diabetes. These findings suggest that phthalate metabolites were powerful predictors of cardiovascular mortality and suggest the potential benefits of maintaining reduced phthalates intake status in reducing premature death in patients with DM.

## Data availability statement

The original contributions presented in the study are included in the article/Supplementary material, further inquiries can be directed to the corresponding authors.

## Ethics statement

The studies involving human participants were reviewed and approved by National Center for Health Statistics (NCHS) Research Ethics Review Board (ERB). The patients/participants provided their written informed consent to participate in this study.

## Author contributions

ZW, YD, SG, GH, and XG conceived the study, performed manuscript revision, and took accountabilities for all aspects of the work. ZW, YD, ZL, ZZ, QF, MZ, TS, GH, and XG performed the data interpretation and drafted and revised the manuscript. ZW, YD, and SG designed the methodology and did the software analysis. GH and XG were in charge of supervision and administration. All authors contributed to the article and approved the submitted version.

## Funding

This work was supported by the Xiangshan Talented Scientific Research Project of Zhuhai People’s Hospital (2021XSYC-03).

## Conflict of interest

The authors declare that the research was conducted in the absence of any commercial or financial relationships that could be construed as a potential conflict of interest.

## Publisher’s note

All claims expressed in this article are solely those of the authors and do not necessarily represent those of their affiliated organizations, or those of the publisher, the editors and the reviewers. Any product that may be evaluated in this article, or claim that may be made by its manufacturer, is not guaranteed or endorsed by the publisher.
