# Supplementary material for: Association of urinary phthalate metabolites with all-cause and cardiovascular disease mortality among adults with diabetes mellitus: National Health and Nutrition Examination Survey 2005–2014
Source: Front Public Health. 2023 May 30;11:1178057. doi: 10.3389/fpubh.2023.1178057 (PMC10268004; doi:10.3389/fpubh.2023.1178057)
Supplement: Supplementary file 1 [file Table_1.DOCX]

**Supplement Table 1 Associations of phthalate concentrations with all-cause and cardiovascular disease mortality among participants with non-DM.**

|  | **All-cause mortality of HR (95% CI) among non-DM** | **Cardiovascular disease mortality of HR (95% CI) among non-DM** |
| --- | --- | --- |
| MCNP*^*^*, μg/g | 1.05 (0.91-1.21) | 1.12 (0.80-1.57) |
| MCOP*^*^*, μg/g | 1.02 (0.87-1.20) | 1.01 (0.74-1.37) |
| MnBP*^*^*, μg/g | 1.09 (0.93-1.28) | 1.22 (0.79-1.88) |
| MEP*^*^*, μg/g | 1.06 (0.98-1.14) | 1.01 (0.83-1.23) |
| MBzP*^*^*, μg/g | 1.14 (0.98-1.33) | 1.17 (0.83-1.67) |
| MCPP*^*^*, μg/g | 1.02 (0.90-1.15) | 0.92 (0.66-1.29) |
| MEHHP*^*^*, μg/g | 1.05 (0.91-1.21) | 1.03 (0.72-1.46) |
| MEOHP*^*^*, μg/g | 1.04 (0.91-1.21) | 1.05 (0.74-1.50) |
| MiBP*^*^*, μg/g | 1.01 (0.85-1.20) | 0.99 (0.71-1.37) |
| MECPP*^*^*, μg/g | 1.09 (0.95-1.25) | 1.18 (0.87-1.60) |
| ∑DEHP*^*^*, μmol/g | 1.07 (0.92-1.24) | 1.10 (0.78-1.56) |

Abbreviations: DM: diabetes mellitus; HR: hazard ratio; CI: confidence interval.

Adjusted for sex, age, ethnicity group, education, family income, the survey cycle, smoking status, drinking status, physical activity, BMI, hypertension, total cholesterol, and family history of cardiovascular diseases.
